# Breeding crops for drought-affected environments and improved climate resilience

**DOI:** 10.1093/plcell/koac321

**Published:** 2022-11-12

**Authors:** Mark Cooper, Carlos D Messina

**Affiliations:** Queensland Alliance for Agriculture and Food Innovation (QAAFI), The University of Queensland, Brisbane, Queensland 4072, Australia; ARC Centre of Excellence for Plant Success in Nature and Agriculture, The University of Queensland, Brisbane, Queensland 4072, Australia; Horticultural Sciences Department, University of Florida, Gainesville, Florida 32611, USA

## Abstract

Breeding climate-resilient crops with improved levels of abiotic and biotic stress resistance as a response to climate change presents both opportunities and challenges. Applying the framework of the “breeder’s equation,” which is used to predict the response to selection for a breeding program cycle, we review methodologies and strategies that have been used to successfully breed crops with improved levels of drought resistance, where the target population of environments (TPEs) is a spatially and temporally heterogeneous mixture of drought-affected and favorable (water-sufficient) environments. Long-term improvement of temperate maize for the US corn belt is used as a case study and compared with progress for other crops and geographies. Integration of trait information across scales, from genomes to ecosystems, is needed to accurately predict yield outcomes for genotypes within the current and future TPEs. This will require transdisciplinary teams to explore, identify, and exploit novel opportunities to accelerate breeding program outcomes; both improved germplasm resources and improved products (cultivars, hybrids, clones, and populations) that outperform and replace the products in use by farmers, in combination with modified agronomic management strategies suited to their local environments.

## Teaser

We review approaches that have been used to breed crops with improved levels of drought resistance.

## Introduction: breeding for drought resistance within agricultural environments

Breeding crops with improved levels of abiotic and biotic stress resistance, as a response to predicted elevated levels of the environmental stresses associated with the effects of climate change, presents both opportunities and challenges if we are to develop sustainable agricultural systems for an uncertain future (Brummer et al., 2011; Chapman et al., 2012; Levin et al., 2012; Fischer et al., 2014; Wang et al., 2015; Rodell et al., 2018; Ceccarelli and Grando, 2020a; Cooper et al., 2021a; Kholová et al., 2021; Langridge et al., 2021; Li et al., 2021; The Rockefeller Foundation, 2021; Messina et al., 2022c). The occurrence of plant abiotic stress in agricultural environments is more often the rule than the exception (Cooper and Hammer, 1996; Chapman et al., 2000; Blum, 2011a; Chenu et al., 2011; Ray et al., 2015; Cooper et al., 2020). This situation is expected to increase in frequency for many regions due to the effects of climate change (Ceccarelli et al., 2010; Chapman et al., 2012; Harrison et al., 2014; Lobell et al., 2015; Rodell et al., 2018; Hammer et al., 2020; Ceccarelli and Grando, 2020a; Cooper et al., 2021a). While water limitations can be a major contributor to the abiotic stress conditions encountered, there are often additional abiotic and biotic factors that add to and interact with the water limitations, contributing to the impact of the imposed stress conditions and yield reductions (Ceccarelli and Grando, 2020a). For the major crops in many of the world’s agricultural regions, inter-annual climate variability contributes substantially to crop yield variability (Ray et al., 2015). This source of crop yield variability is expected to increase further under the pressures of climate change (Chapman et al., 2012; Langridge et al., 2021). Reduced agricultural productivity has been documented from a range of abiotic environmental stresses that are consequences of water limitations occurring during the crop lifecycle in many of the world’s agricultural systems; we refer to these as water-limited environments (Blum, 2011a; Chenu et al., 2011; Boyer et al., 2013; Kholová et al., 2013; Gaffney et al., 2015; Cooper et al., 2020).

Van Ittersum et al. (2013) suggested using a reference of 80% of the yield potential that could be achieved in the absence of abiotic or biotic limitations to crop productivity as a practical target for designing improved crop management strategies to reduce on-farm yield-gaps; the yield-gap being the difference in yield between the potential crop yield within the environment with no resource limitations and the actual yield that is achieved by the farmer (Fischer et al., 2014). We adopt the same convention with respect to crop breeding for improved levels of drought resistance (Cooper et al., 2020, 2023; Messina et al., 2022a). That is, consideration should be given to targeted breeding to improve drought resistance when the total supply of water (from stored soil moisture, rainfall, and irrigation) available to a crop during its lifecycle is below the crop demand needed to achieve consistent harvestable yields of at least 80% of the potential yield in the target agricultural environment (Ludlow and Muchow, 1990; Fukai and Cooper, 1995; Campos et al., 2004; Messina et al., 2011, 2022a, 2022b; Van Ittersum et al., 2013; Cooper et al., 2014a, 2020, 2021a, 2021b, 2023; Fischer et al., 2014; Gleason et al., 2022). Here, we consider trait networks to be coordinated combinations of multiple traits, which together operate in ways that contribute to enhanced responses of crops to specific environments, or a range of environmental conditions that occur within a target population of environments (TPEs), beyond the responses that can be achieved by the individual traits operating in isolation (Hammer et al., 2006; Cooper et al., 2009, 2021a; Tardieu et al., 2021; Gleason et al., 2022). One of the opportunities we consider herein is the development of crop growth models as both a framework and an enabling transdisciplinary tool to investigate traits and trait networks and their potential for applications to accelerate plant breeding for drought resistance and to enhance trait discovery contributions to improved climate resilience (Cooper and Hammer, 1996; Hammer et al., 2006, 2019).

**Figure 1:**
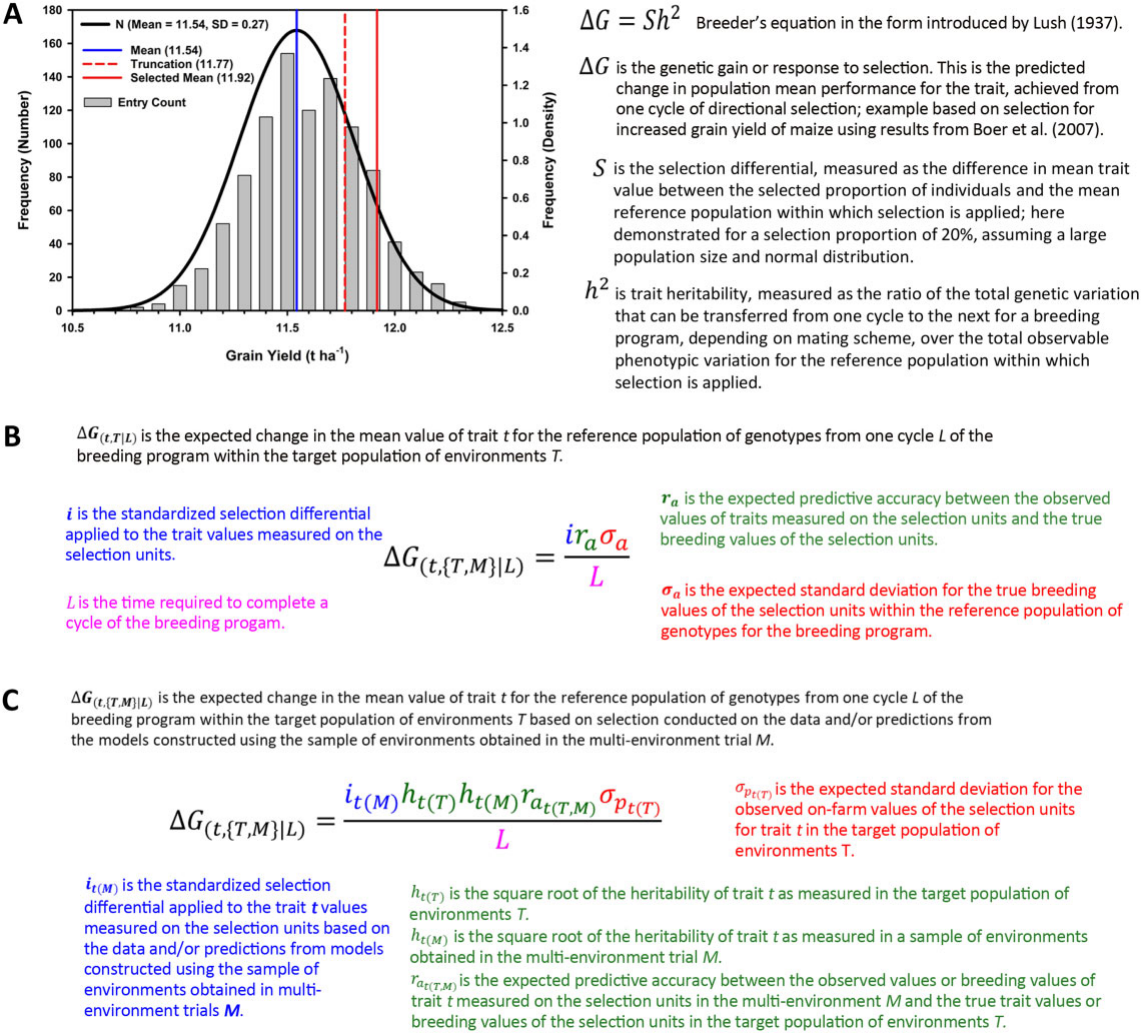
Three formulations of the breeder’s equation. A, The formulation first introduced by Lush (1937), together with a graphical representation based on the grain yield results reported by Boer et al. (2007) for a maize multi-environment trial (MET) conducted to evaluate a sample of progeny from a large biparental mapping study within the US corn belt. B, The second form is structured to emphasize the connection between the genetic variation for breeding values among individuals in the reference population of genotypes and the predictive accuracy for transmission, from one breeding cycle to the next, of the favorable alleles of the genes controlling the breeding value for a trait. The predictive accuracy component (*r*_a_) of this form of the equation is a research target for improvement through the design of improved G2P models for the application of genomic prediction in plant breeding. C, The third form is structured to emphasize the genetic correlation between the genetic variation for a target trait that can be exposed in METs conducted at stages of a breeding program (e.g. Figures 2 and 4) and the expected trait genetic variation within the TPEs.

Defining and characterizing the TPE for the agricultural systems within which a breeding program operates is foundational to the effective design of breeding programs (Cooper and DeLacy, 1994; Cooper and Hammer, 1996; Chapman et al., 2000; Chenu et al., 2011; Cooper et al., 2014a, 2014b, 2020, 2021a; Kholová et al., 2021; Resende et al., 2021). The on-farm agricultural environments of the TPE are an outcome of the combined effects of the biophysical environment (***E***; soils and climate), and the agronomic management strategies (***M***; crop rotations, planting dates and densities, row spacings, irrigation, fertilizer applications, disease, and pest control measures, mechanization, etc.) that are adopted by farmers for their local environments. The combined influences of both the ***E*** and ***M*** dimensions of the agricultural environment on crop water availability must be considered in breeding for drought resistance. Genetic improvement (***ΔG***; as predicted by the “breeder’s equation” is targeted at the combined environment–management (***E***x***M***) conditions of the agricultural environment and thus must deal with the opportunities and complexities of genotype-by-environment-by-management (***G***x***E***x***M***) interactions (Figure 1; Cooper and Hammer, 1996; Lynch and Walsh, 1998; Cooper et al., 2001, 2020, 2021a, 2023; Messina et al., 2009; Walsh and Lynch, 2018; Irmak et al., 2019; Peng et al., 2020; Hunt et al., 2021; Kholová et al., 2021; Zhao et al., 2022). Furthermore, whenever genotype-by-management interactions are important for effective crop water use for agricultural environments, this may require optimization and targeted improvement of genotype–management (***G–M***) technologies and focused attention on the influence of ***(G–M)xE*** interactions for current and future environments (Duvick et al., 2004; Hammer et al., 2014, 2020; Gaffney et al., 2015; Irmak et al., 2019; Cooper et al., 2020, 2021a, 2023; Peng et al., 2020; Hunt et al., 2021; Messina et al., 2022a, 2022c; Zhao et al., 2022).

Discovery strategies and methodologies for identifying traits and networks of traits contributing to on-farm drought resistance, as targets to accelerate the improvement of crop yield performance, have received significant attention from a range of discipline-centered perspectives; including molecular biology and plant physiology (Blum, 1988, 2011a; Ludlow and Muchow, 1990; Fukai and Cooper, 1995; Campos et al., 2004; Ribaut, 2006; Cattivelli et al., 2008; Messina et al., 2011; Jordan et al., 2012; Mace et al., 2012; Borrell et al., 2014; Guo et al., 2014; Luo et al., 2019; Liedtke et al., 2020; Liu and Qin, 2021; Simmons et al., 2021; Gleason et al., 2022; Welcker et al., 2022). Often this has been done without a clear definition of drought within the target drought-affected agricultural environments of the TPE. The crop physiological framework used to investigate potential contributions of traits to crop performance within water-limited environments for agricultural systems has been continually refined with experience (Sinclair, 2000, 2011; Passioura, 2006; Blum, 2009, 2011a, 2011b; Hammer et al., 2009, 2020; Messina et al., 2009, 2011, 2015; Cooper et al., 2014a; 2020; Gleason et al., 2022). Furthermore, improved drought resistance recently has received renewed emphasis as an important target to develop climate-resilient crops (Reynolds, 2010; Blum, 2011a; Chapman et al., 2012; Ray et al., 2015; Hammer et al., 2021; Langridge et al., 2021; Messina et al., 2022a, 2022c).

**Figure 2:**
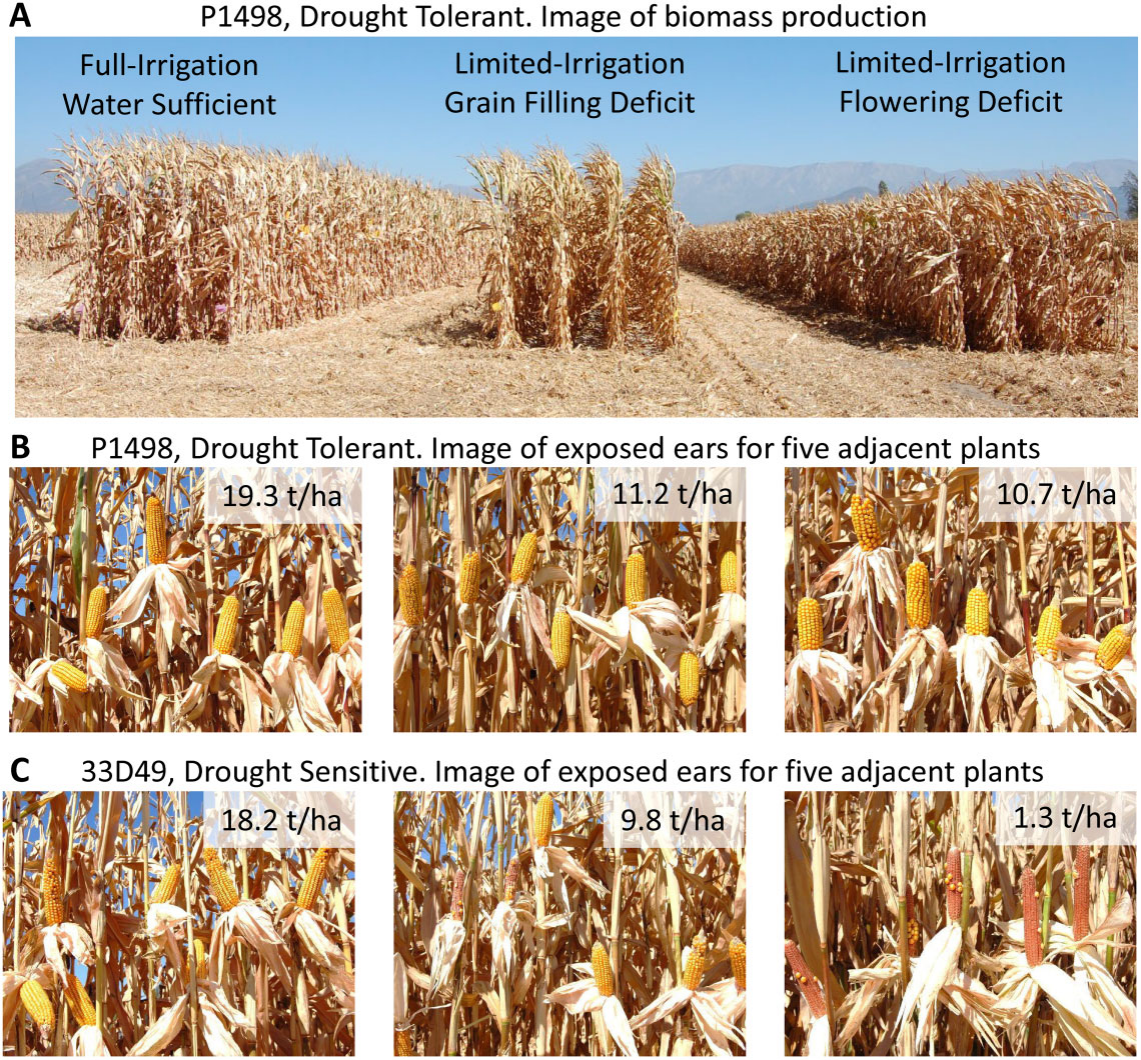
Experimental demonstration of contrasting grain yield reaction-norms for two maize hybrids for a sequence of three environments contrasting in water availability. Images are from the evaluation of two commercial maize hybrids, P1498 (tolerant) and 33D49 (sensitive), with similar yield potential in environments with sufficient water and with contrasting yield responses to water limitations imposed to coincide with the flowering period and post-flowering during the grain-filling period. The experiment was conducted under conditions with no rainfall during the growing season and water was supplied by drip-tape irrigation. A, Above-ground biomass production at the time of harvest for hybrid P1498 in side-by-side plots imposing three levels of irrigation treatment. B, Ears of hybrid P1498 for five adjacent plants within a plot row for each of the three irrigation treatments. C, Ears of hybrid 33D49 for five adjacent plants within a plot row for each of the three irrigation treatments. Yield levels for each hybrid treatment combination were obtained from combined harvest of the whole experimental plot used for the ear images.

There has been much investigation of the physiological basis of traits contributing to measures of plant drought resistance, including drought escape, avoidance, and tolerance and the genetics of specific trait adaptations for water-limited environments, the potential benefits from many of these trait discovery efforts have only been comprehensively evaluated for their realized contributions to accelerated genetic improvement across cycles of breeding programs in a few cases (Figure 2; Blum, 1988, 2011a; Ludlow and Muchow, 1990; Fukai and Cooper, 1995; Chapman et al., 2003; Campos et al., 2004; Barker et al., 2005; Hammer et al., 2006, 2009; Jordan et al., 2012; Mace et al., 2012; Tardieu, 2012, 2022; Cooper et al., 2014a; Guo et al., 2014; Messina et al., 2011, 2015, 2019, 2021, 2022a, 2022b, 2022c; Gaffney et al., 2015; Nuccio et al., 2018; McFadden et al., 2019; Voss-Fels et al., 2019b; Simmons et al., 2021; Tardieu et al., 2021; Nurmberg et al., 2022; Schussler et al., 2022; Welcker et al., 2022; Zhao et al., 2022). The aim of this review is to advance concepts that promote the multidisciplinary dialog and transdisciplinary efforts that are required to evaluate traits and trait network combinations for their impact within a breeding program context. Attention is also given to: (i) the definition of the breeder’s equation framework and some applications that are relevant to breeding for drought resistance and climate resilience, (ii) refining the terminology that is used in relation to drought resistance research as it applies to breeding for the effective use of water within agricultural environments, and (iii) the potential for novel pathways to improve on-farm crop productivity that utilize opportunities from coordinated contributions of improved genetics from breeding, biological understanding of traits from genes to ecosystems, and agronomic management.

## The breeder’s equation framework: a foundation for applications of physiological knowledge to breeding for drought resistance and climate resilience

There are many ways to model plant breeding programs to evaluate their potential for breeding across cycles toward improved performance for specific targets, such as improved drought resistance, and to accelerate crop genetic improvement for the TPE of an agricultural system. The “breeder’s equation” (Figure 1) and its many extensions provide a useful breeding-focused prediction framework that will be used herein as a foundation for the considerations of trait genetics, trait networks, and trait ecophysiology to breed crops for drought resistance. For historical origins, theoretical developments and successful applications of the breeder’s equation, see Lush (1937), Comstock (1996), Lynch and Walsh (1998), Podlich and Cooper (1998), Chapman et al. (2003), Messina et al. (2011), Cooper et al. (2014a, 2021b), Walsh and Lynch (2018), Araus et al. (2018), Voss-Fels et al. (2019a), Cobb et al. (2019), Kholová et al. (2021), and Technow et al. (2021). While there are many detailed formulations of the breeder’s equation, the basic structure predicts the expected change in the mean value of trait *t* (***ΔG*_(*t*|L)_**; also, often referred to as the response to selection or genetic gain) within the TPE for the reference population of genotypes of the breeding program that can be achieved from a cycle *L* of the breeding program. The response to selection for the trait is predicted as the product of three components relative to the time taken to complete a cycle *L* of the breeding program (Figure 1). The three components are: (1) the selection pressure *i_t_* applied to change the mean value of a trait, implemented by culling inferior individuals from the reference population and identifying the individuals retained in the breeding program and used to generate, through an organized mating scheme, the individuals of the next cycle of the breeding program, (2) the trait heritability $h_{t}^{2}$ or predictive accuracy $r_{a_{t}}$, which provides a measure or prediction of the fraction of the total observable trait variation (via direct observation or a suitable genome-to-phenome (G2P) model for traits, discussed further below), to which the selection pressure is applied, that can be transferred via transmission of the alleles of the genes controlling the trait through the structured mating scheme from one cycle of the breeding program to the next, and (3) the observable trait variation for the trait within the reference population of genotypes for the breeding program. Over repeated cycles of the breeding scheme, it is expected that the favorable alleles of the genes controlling the target trait will increase in frequency in the reference population of genotypes (Comstock, 1996; Lynch and Walsh, 1998; Cooper et al., 2014a; Walsh and Lynch, 2018; Wisser et al., 2019; Powell et al., 2022). In response to their increase in frequency, the mean value of the trait is predicted to increase in the reference population, as observed for long-term maize breeding (Duvick et al., 2004; Cooper et al., 2014a, 2014b; Technow et al., 2021).

Underpinning the mechanistic model of the breeder’s equation (Figure 1) is the G2P model that connects the trait genetic variation at the level of the allelic variation for the genes at positions within the genome with the observable trait phenotypic variation at the level of the individuals that comprise the reference population of genotypes. For quantitative genetics, the core G2P model for traits is commonly referred to as the infinitesimal model (Lynch and Walsh, 1998; Walsh and Lynch, 2018). The basic premise of the infinitesimal model assumes that there are a large number of genes, regulatory regions, and a range of genome structural variants, in the order of thousands, distributed throughout the genome, each with allelic variation that influences the trait phenotypic variation among individuals within the reference population of genotypes. Applying statistical estimation methods to appropriately designed experiments, plant breeders can estimate the elements of the breeder’s equation (Figure 1) to predict expected response to selection and obtain estimates of positions of the genome that are involved in the G2P model (Yu and Buckler, 2006; Yu et al., 2006, 2008; Boer et al., 2007; Buckler et al., 2009; Zhang et al., 2010; Wisser et al., 2019; Diepenbrock et al., 2022). While for a few traits of importance, G2P models based on a small number of large effect genes have been indicated, the most common result for complex traits such as yield and drought resistance is the indication that large numbers of small effect genes control the outcome of these traits, as approximated by the infinitesimal model (Boer et al., 2007; Buckler et al., 2009; Cooper et al., 2014a, 2014b; Wisser et al., 2019; Diepenbrock et al., 2022). This insight emerging from the use of the infinitesimal model in predictive breeding should be of interest to plant and crop physiologists investigating the mechanistic and ecophysiological bases of the G2P relationships for the traits controlling plant growth and development within environments. Results from the use of predictive algorithms based on the infinitesimal model provide a relevant experimental control against which the utilization of any additional prior biological knowledge used to predict G2P relationships can be judged for its merits in improving the opportunities for a plant breeder to predict response to selection for breeding objectives (Figure 1; Jackson et al., 1996; Cooper et al., 2005, 2021b; Messina et al., 2009; Technow et al., 2015; Araus et al., 2018; Hammer et al., 2019; Powell et al., 2021, 2022; Diepenbrock et al., 2022). In this review, we apply the breeder’s equation within this framework to discuss the use of molecular and physiological knowledge of traits for any applications to accelerate breeding for drought resistance and improved climate resilience (Chapman et al., 2012; Voss-Fels et al., 2019a; Langridge et al., 2021).

The basic structure of the breeder’s equation can be extended in many ways to accommodate information for multiple traits and trait networks and the explicit incorporation of genes and gene networks that control the traits and details of the G2P mapping for the traits (Figure 1; Chapman et al., 2003; Cooper et al., 2005, 2009, 2021b; Hammer et al., 2006; Messina et al., 2011; Walsh and Lynch, 2018; Bustos-Korts et al., 2019a, 2019b, 2021; Technow et al., 2021; Diepenbrock et al., 2022; Powell et al., 2022). For a range of breeding program designs and quantitative genetic models of trait G2P architectures, explicit forms of the breeder’s equation have been defined (Hallauer and Miranda, 1988; Lande and Thompson, 1990; Comstock, 1996). However, with the advances in computer simulation capabilities, it is now possible to model response to selection for any breeding program design for individual and multiple cycles using diverse sources of G2P trait knowledge and data structures (Podlich and Cooper, 1998; Chapman et al., 2003; Campos et al., 2004; Messina et al., 2011; Jahufer and Luo, 2018; Voss-Fels et al., 2019a; Bernardo, 2020; Cooper et al., 2021b; Gaynor et al., 2021; Technow et al., 2021; Powell et al., 2022).

## Foundations: historical improvements in breeding for drought resistance

When considering breeding for drought resistance and its contributions to improved crop climate resilience for predicted climate change scenarios, it is important to understand the progress that has been achieved to date; considering both what has worked and what has not (Figure 2; Campos et al., 2004; Duvick et al., 2004; Barker et al., 2005; Blum, 2011a; Cooper et al., 2014a, 2014b; Guo et al., 2014; Nuccio et al., 2018; Voss-Fels et al., 2019a; Simmons et al., 2021; Technow et al., 2021; Messina et al., 2022a; Welcker et al., 2022). Through the sustained efforts of long-term breeding programs, by creating a progression of improved genotypes that were released over multiple breeding cycles, improvements in on-farm crop productivity have been achieved for multiple crops and a diverse range of target water-limited environments (Figure 2; Duvick et al., 2004; Hammer et al., 2009; Blum, 2011a; Jordan et al., 2012; Cooper et al., 2014a, 2014b; Fischer et al., 2014; Richards et al., 2014; Snowdon et al., 2020; Prasanna et al., 2021; Xiong et al., 2021; Messina et al., 2022a; Nurmberg et al., 2022; Welcker et al., 2022). However, it is recognized that many challenges remain if we are to retain and build on the progress that has been made and target further improvements for the future complex TPE expected under climate change scenarios (Brummer et al., 2011; Chapman et al., 2012; Fischer et al., 2014; Rodell et al., 2018; Ceccarelli and Grando, 2020a; Cooper et al., 2021a; Kholová et al., 2021).

Large on-farm yield-gaps exist and persist for many water-limited regions of the world, requiring renewed consideration of crop improvement strategies that integrate breeding efforts with agronomic management research that are grounded on a biological understanding of plant adaptation and the components of the strategies that have worked (Cooper and Hammer, 1996; Campos et al., 2004; Barker et al., 2005; Lobell et al., 2009; Van Ittersum et al., 2013, 2016; Hammer et al., 2014; Van Bussel et al., 2015; Gaffney et al., 2015; Hatfield and Walthall, 2015; Irmak et al., 2019; Cooper et al., 2020, 2021a; Peng et al., 2020; Hunt et al., 2021; Zhao et al., 2022). Furthermore, the multiple influences of climate change and the consequences of any associated increased environmental variability on crop performance will necessitate a definition and quantification of how the TPE is changing; and more importantly, the rate at which these breeding targets are changing will determine the capacity for improving crop adaptation in response to climate change (Braun et al., 2010; Chapman et al., 2012; Harrison et al., 2014; Lobell et al., 2015; Rodell et al., 2018; Ceccarelli and Grando, 2020a; Hammer et al., 2020; Snowdon et al., 2020; Cooper et al., 2021a; Xiong et al., 2021; Messina et al., 2022a, 2022c). Here, we review examples of progress that has been achieved through targeted breeding for drought resistance to water-limited environments and consider their contributions to the current levels of climate resiliency of agricultural systems and the needs and prospects for the future. We draw on our experiences from breeding maize hybrids for the mixture of water-limited (dryland-rainfed and limited-irrigation) and water-sufficient (rainfall supplemented with unlimited-irrigation) environments in the Western region of the US corn belt and consider similarities and differences with experiences reported for other crops and regions (Campos et al., 2004; Barker et al., 2005; Löffler et al., 2005; Blum, 2011a; Jordan et al., 2012; Cooper et al., 2014a, 2014b; Lobell et al., 2014; Richards et al., 2014; Gaffney et al., 2015; Adee et al., 2016; McFadden et al., 2019; Kholová et al., 2021; Prasanna et al., 2021; Diepenbrock et al., 2022; Mayor et al., 2022; Messina et al., 2022a, 2022c; Nurmberg et al., 2022; Welcker et al., 2022).

Successful plant breeding and breeding methodology refinement focus on the elements of the breeder’s equation (Figure 1) to optimize a systematic breeding program that is designed to enable efficient use of the available genetic, genomic, and germplasm resources and trait genetic information to achieve short, medium, and long-term product development goals over multiple breeding program cycles (Campos et al., 2004; Duvick et al., 2004; Barker et al., 2005; Hammer et al., 2009; Cooper et al., 2014a, 2014b; Cobb et al., 2019; Voss-Fels et al., 2019a; Kholová et al., 2021; Technow et al., 2021; Covarrubias-Pazaran et al., 2022). A breeding program is designed to deliver new products (populations, cultivars, clones, or hybrids, depending on the species) to meet specified target product profiles (Duvick et al., 2004; Cobb et al., 2019; Kholová et al., 2021). These product profiles are defined through continuous interaction with farmers; whether they operate subsistence or large commercial farms (Duvick et al., 2004; Blum, 2011a; Cooper et al., 2014a; Gaffney et al., 2015; Ceccarelli and Grando, 2020a, 2020b; Kholová et al., 2021; Covarrubias-Pazaran et al., 2022). Furthermore, a breeding program is designed to operate over multiple cycles for the evolving TPE of an agricultural system (Duvick et al., 2004; Voss-Fels et al., 2019a; Snowdon et al., 2020; Messina et al., 2022a, 2022c). Consequences of climate change for breeding include changes in the environmental composition of the TPE and refinements of the desired target product profiles required by the customers. The same considerations apply to breeding for drought resistance and its contributions to climate resilience (Chapman et al., 2012; Cooper et al., 2014a, 2014b; Gaffney et al., 2015; Messina et al., 2022a, 2022c).

There have been many proposals to use combinations of genomic technologies and trait understanding across biological scales to improve the efficiency of breeding programs and to accelerate outcomes from breeding programs (Blum, 1988, 2011a; Jackson et al., 1996; Meuwissen et al., 2001; Hammer et al., 2006; Tester and Langridge, 2010; Bailey-Serres et al., 2019; Voss-Fels et al., 2019a; Simmons et al., 2021; Varshney et al., 2021a, 2021b, 2021c). Their adoption and impact have been variable (Bernardo, 2016). For some proposals, it is too early to assess their impact and full potential to enhance breeding for drought resistance and climate resilience. Genomic prediction has demonstrated widespread potential to accelerate rates of genetic progress from crop breeding. It has been successfully implemented into large-scale commercial maize breeding programs and is under a wide range of stages of evaluation and adoption for other crops, regions, and scales of breeding program (Meuwissen et al., 2001; Cooper et al., 2014a, 2014b; Voss-Fels et al., 2019a; Velazco et al., 2019; Varshney et al., 2021a, 2021b, 2021c). Refinements of the basic form of the breeder’s equation (Figure 1) provide a foundation for considering novel applications of genomic prediction methodologies to breeding for improved levels of drought resistance and climate resilience. These will be discussed further below.

The term drought is widely used to refer to a severe deficit of available water relative to crop requirements, at one or more stages during the crop lifecycle, which can have a negative impact on crop productivity and quality (Figure 2; Campos et al., 2004; Passioura, 2006; Blum, 2009, 2011a, 2011b; Sinclair, 2011; Boyer et al., 2013; Lobell et al., 2014; Gaffney et al., 2015). To understand the impact of water availability on the occurrence of drought within the agricultural environment, to enable breeding for drought resistance, and in turn to understand the impact of any changes in crop productivity on the sustainability of the current and future agricultural systems, characterizing the quantity and timing of water availability, relative to crop requirements throughout the crop lifecycle for agricultural systems, is a foundational requirement (Chapman et al., 2000; Rosegrant et al., 2009; Blum, 2011a; Chenu et al., 2011; Boyer et al., 2013; Kholová et al., 2013; Ray et al., 2013, 2015; Cooper et al., 2014a, 2014b; Lobell et al., 2014; Van Ittersum et al., 2016; Rodell et al., 2018; Washburn et al., 2021; Gleason et al., 2022; Messina et al., 2022a, 2022c).

To target breeding for water-limited environments, utilizing contributions from drought resistance traits (escape, avoidance, and tolerance) and trait networks, it is important to distinguish between water deficits that reduce crop yield while still enabling sufficient yield levels to remain above the threshold for viable agricultural productivity, which we will refer to as agricultural drought, and the catastrophic, severe water deficits that reduce yield below the viability threshold but still allow plant survival, which we will refer to as survival drought; the threshold that distinguishes between agricultural and survival droughts will vary for different agricultural systems, ranging from small-scale subsistence to large-scale industrial (Ceccarelli, 1994; Cooper and Hammer, 1996; Blum, 2011a; Chenu et al., 2011; Van Ittersum et al., 2013, 2016; Gaffney et al., 2015; Kholová et al., 2021; Tardieu, 2022). Within this review, we focus on enabling and accelerating breeding for yield productivity for agricultural droughts, where crop yields are reduced due to water limitations, but remain above the threshold for viable agricultural productivity (Blum, 2011a; Lobell et al., 2014; Gaffney et al., 2015; Cooper et al., 2020, 2021a, 2023; Kholová et al., 2021; Messina et al., 2022a; Tardieu, 2022). The potential of such breeding strategies, designed to develop products that can help to reduce yield losses from agricultural droughts, to contribute to long-term climate resilience of agriculture will be considered.

## Yield reaction-norms: genotype by environment interactions

**Figure 3:**
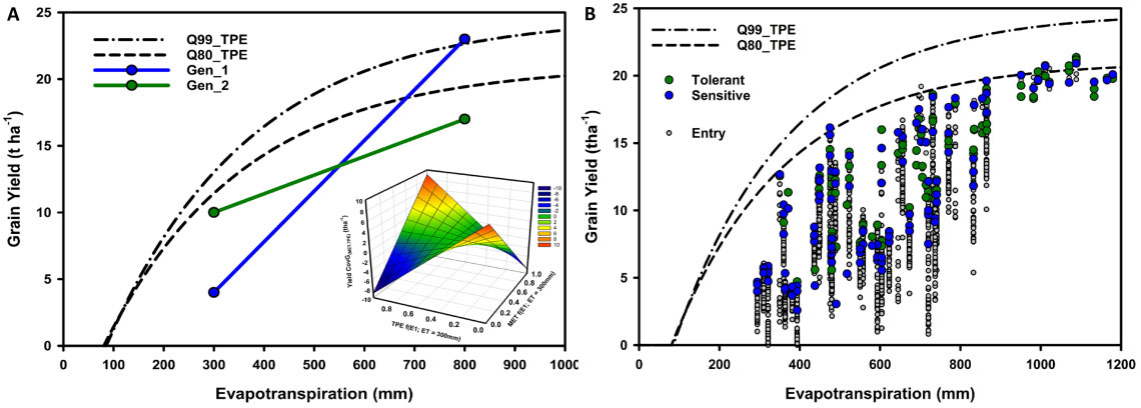
Schematic representations of GxE interactions and contrasting reaction-norms for grain yield of maize hybrids (genotypes) with contrasting levels of drought resistance and yield potential and environments contrasting for water availability, represented by a continuum of crop evapotranspiration. A, Theoretical representation of extreme crossover genotype-by-environment interactions for two genotypes based on contrasting yield-evapotranspiration reaction-norms (Gen_1, high yield potential and drought-sensitive; Gen_2, low yield potential and drought resistant) in response to environmental contrasts in water availability as quantified in terms of season total crop evapotranspiration (Env_1, environment-type characterized by low water availability; Env_2, environment-type characterized by high water availability). The two hybrid grain yield reaction-norms are superimposed on two yield-evapotranspiration fronts estimated by applying quantile regression (Q99%, 99% quantile regression; Q80%, 80% quantile regression) to a large sample of simulated GxExM combinations designed to represent the TPEs of the US corn belt (Cooper et al., 2020). The insert plots the theoretical genetic covariance between the yield variation observed in a breeding MET and the TPEs as the frequency of the two environment types (Env_1, ET = 300 mm and Env_2 = 800 mm) sampled in the MET changes for 0 to 1, relative to their frequency in the TPE, ranging from 0 to 1. The genetic covariance for grain yield between the MET and the TPE is used in combination with the genetic variance within the MET and the TPE to estimate the genetic correlation between the MET and the TPE as represented in form three of the breeder’s equation in Figure 1. B, Empirical grain yield results for a set of maize hybrids evaluated across a range of environments with different levels of water availability as determined by crop evapotranspiration. The empirical results are also superimposed on the Q99% and Q80% yield-evapotranspiration fronts (Cooper et al., 2020). A group of hybrids characterized as drought tolerant, and a group of hybrids characterized as drought sensitive, as depicted in Figure 2, are identified from the full set of hybrid entries in the MET.

The reaction-norm of a genotype is defined as a genotype-specific functional relationship between a trait phenotype(s) (e.g. yield) and an environmental gradient(s) (e.g. evapotranspiration) (Figure 3; Woltereck, 1909; Gregorius and Namkoong, 1986; Boer et al., 2007; Jarquín et al., 2014; Van Eeuwijk et al., 2016; Irmak et al., 2019; Cooper et al., 2021a; Messina et al., 2022a). Breeding for drought tolerance requires consideration of and selection for genotype yield reaction-norms across environments, and therefore the extent and factors contributing to genotype-by-environment (**GxE**) interactions within the TPE; herein, this includes consideration of ***G***x***(E***x***M)*** interactions, as discussed above (Figure 3; Cooper and DeLacy, 1994; Cooper and Hammer, 1996; Basford and Cooper, 1998; Cooper et al., 2001, 2014a, 2014b, 2020, 2021a, 2023; Messina et al., 2009, 2022a, 2022c; Gaffney et al., 2015; Van Eeuwijk et al., 2016; Gage et al., 2021; Li et al., 2021; Diepenbrock et al., 2022). The incidence of GxE interactions for crop productivity traits, such as yield, has long been recognized as a vexing challenge with major implications for many aspects of plant breeding, including trait prediction, selection, and on-farm product performance (Figures 1–3; Haldane, 1946; Falconer, 1952; Comstock and Moll, 1963; Finlay and Wilkinson, 1963; Allard and Bradshaw, 1964; Eberhart and Russell, 1966; Knight, 1970; Ceccarelli, 1989, 1994; Cooper and DeLacy, 1994; Cooper and Hammer, 1996; Blum, 2011a; Gaffney et al., 2015; Van Eeuwijk et al., 2016; Ceccarelli and Grando, 2020a, 2020b; Gage et al., 2021; Li et al., 2021; Messina et al., 2022a; Piepho, 2022). Differences in the form of reaction-norm functions among genotypes provide a framework for studying important properties of GxE interactions within a TPE and their implications for plant breeding and agronomic management strategies designed to reduce the impact of agricultural drought (Hammer et al., 2014, 2019, 2020; Cooper et al., 2020; Hunt et al., 2021).

The influences of GxE interactions can occur at many levels, from gene expression to whole plant trait phenotypes. Within the plant breeding context, they are most frequently considered at the whole plant level and can be defined and studied in terms of significant changes in the relative trait performance of genotypes with changes in environmental conditions, such as water availability (Figures 2 and 3; Haldane, 1946; Ceccarelli, 1989, 1994; Cooper and DeLacy, 1994; Smith et al., 2005; Boer et al., 2007; Blum, 2011a; Van Eeuwijk et al., 2016; Rogers et al., 2021; Nurmberg et al., 2022; Piepho, 2022). Plant breeders typically focus on GxE interactions that diminish the genetic correlation for traits between environments (Figure 1C) and result in changes in the rank of genotypes, as these types of GxE interactions have the greatest potential to complicate the selection decisions made by breeders at the different stages within a breeding program cycle and across cycles of the breeding program (Figure 3A; Haldane, 1946; Falconer, 1952; Knight, 1970; Cooper and DeLacy, 1994; Podlich et al., 1999; Blum, 2011a; Voss-Fels et al., 2019a; Smith et al., 2021a; Xiong et al., 2021). In the case of breeding for drought resistance, the focus is on partitioning the total environment (***ExM***) dimension into informative and measurable components that are relevant for analyzing genotype responses to the continuum of water availability, ranging from drought to water sufficiency (Figures 2 and 3; French and Schultz, 1984; Chapman et al., 2000; Campos et al., 2004; Blum, 2011a; Chenu et al., 2011; Cooper et al., 2020, 2021a; Cooper and Messina, 2021; Nurmberg et al., 2022; Welcker et al., 2022). To assess the value of targeted breeding for drought resistance, it is important to quantify the magnitude of any drought-specific GxE interaction components as part of the total pool of GxE interactions for the TPE (Chapman et al., 2000, 2003, 2012; Blum, 2011a; Chenu et al., 2011; Messina et al., 2011; Kholová et al., 2013; Resende et al., 2021; Messina et al., 2022a). Further, changes in the importance of drought and other environmental stresses with time, in response to the effects of climate change and their potential multiple influences on patterns of GxE interactions, require consideration to enable the design and optimization of breeding programs conducted over multiple cycles (Messina et al., 2011, 2022a; Chapman et al., 2012; Harrison et al., 2014; Lobell et al., 2015; Voss-Fels et al., 2019a; Peng et al., 2020; Snowdon et al., 2020; Cooper et al., 2021a; Xiong et al., 2021). The networks of traits contributing to improved yield reaction-norms for the TPE can be expected to change with the multiple effects of climate change on the environmental composition of the TPE (Messina et al., 2011, 2015, 2022a, 2022b; Chapman et al., 2012; Cooper et al., 2021a; Li et al., 2021; Gleason et al., 2022). The challenge for the design of the required multi-disciplinary research programs to be conducted by breeders, geneticists, physiologists, and agronomists is to chart these environmental changes and propose promising multiple, workable ***(GxM)xE*** solutions that can be evaluated and further improved over cycles of the breeding program as the environmental composition of the TPE shifts due to the effects of climate change (Voss-Fels et al., 2019a; Snowdon et al., 2020; Cooper et al., 2021a, 2021b, 2023; Technow et al., 2021).

## Targeted breeding for drought resistance: enviromics and envirotyping

**Figure 4:**
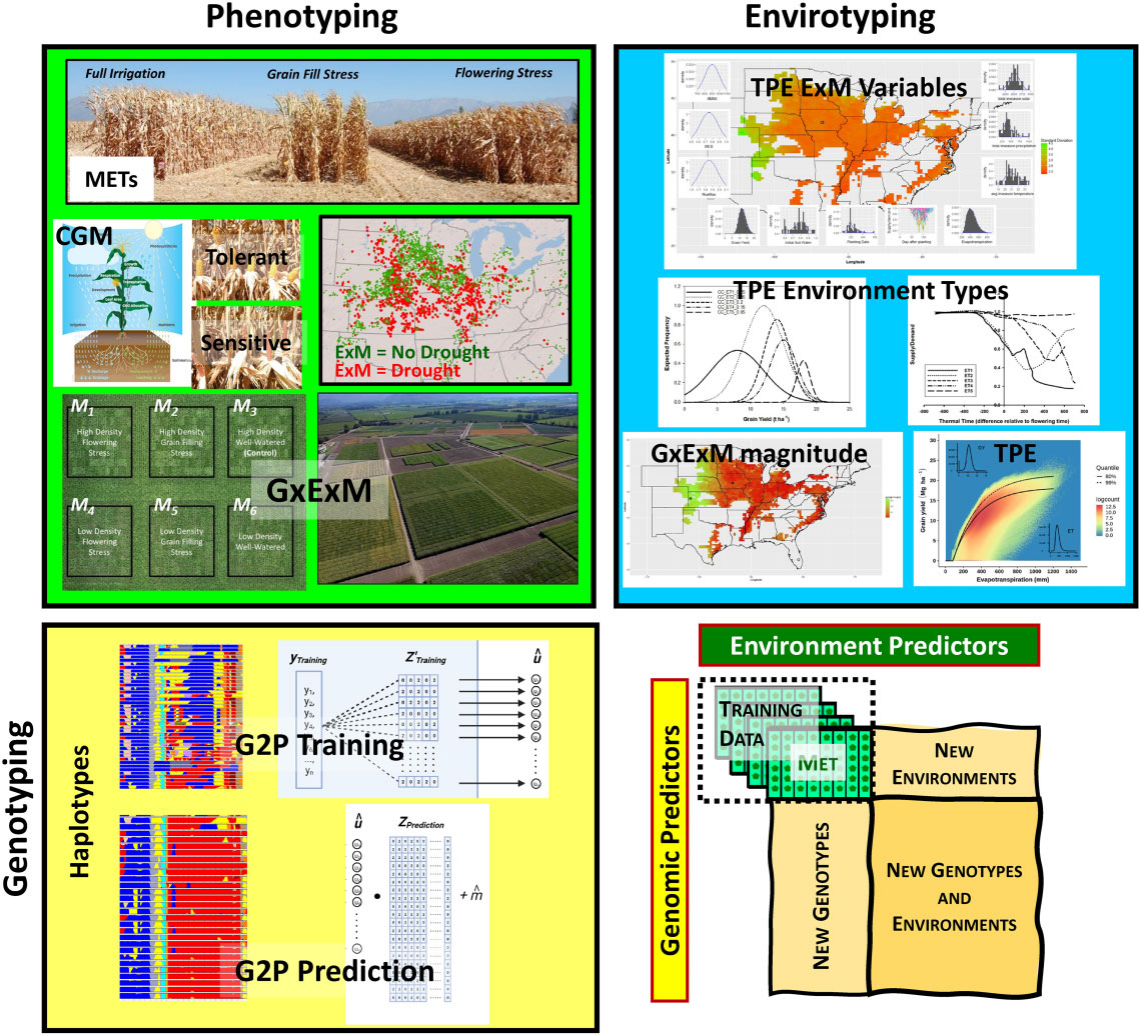
Integration of phenotyping, genotyping, and envirotyping to create training data sets for breeding prediction applications. Different views of key components and methodologies contributing to phenotyping, envirotyping, and genotyping activities involved in the conduct of breeding METs for stages of a plant breeding program. The accumulation of MET data sets over multiple breeding program cycles can be used to design appropriate training data sets to develop models for genomic prediction applications in breeding. The genotyping of individual entries is used to construct genotypic predictors based on individual markers (e.g. single-nucleotide polymorphisms; SNPs) or combinations of contiguous markers used to form haplotypes. The envirotyping activities are undertaken to construct enviromic predictors used to distinguish the different characteristics of the environments (e.g. crop evapotranspiration to integrate many environmental and crop variables that determine the availability of water to the crop and distinguish between water-limited and water-sufficient environments).

To chart the rate and trajectory of environmental changes within a TPE, as they unfold with climate change, we require improved technologies to characterize agricultural environments. Enviromics for breeding applications refers to the collection of activities that use measurements of biophysical environmental variables to characterize the environmental conditions that influence crop performance, GxE interactions, and differences among the reaction-norms of genotypes (Figures 2–4; Cooper and Hammer, 1996; Chapman et al., 2000; Blum, 2011a; Chenu et al., 2011; Van Eeuwijk et al., 2016; Xu, 2016; Millet et al., 2019; Cooper and Messina, 2021; Resende et al., 2021, 2022; Diepenbrock et al., 2022). Envirotyping, or grouping environments in terms of the repeatable sets of variables that impact genotype performance, can be applied at any stage of a breeding program, from controlled environments to on-farm testing (Figure 4; Cooper et al., 2014a, 2014b; Gaffney et al., 2015; Messina et al., 2015; Resende et al., 2021; Langstroff et al., 2022). Breeding for drought environment-type targets enables focused drought breeding strategies with improved resolution that goes beyond the common extremes of breeding for broad adaptation across the whole TPE or breeding for specific adaptation to every farmer’s field. For example, the environment types can be used to target breeding efforts for the gradient of water availability expected in the TPE (Figure 3) to focus on the combinations of environmental and management conditions that result in drought impacting crop performance at different stages of development, such as flowering and grain-filling (Figure 2). As depicted in Figure 4, genomic and enviromic predictors can be used in combination with the G2P model developed using training data sets to predict the yield performance of genotypes for different environments. Beyond the bounds of the training data set, three classes of application are of interest: (1) predicting the performance of new genotypes created in the breeding program that have not been phenotyped into the same environmental conditions sampled in the training data set, (2) predicting the performance of the genotypes evaluated within the training data set to new environmental conditions, as expected under the influences of climate change, that were not included in the training data set, and (3) predicting the performance of the new genotypes in new environmental conditions. The third case has direct relevance to breeding through prediction methodology for future scenarios expected under the influences of climate change.

Envirotyping can be used to investigate the relationships between the environments that are sampled in the multi-environment trials designed to test genotypes at the different stages of breeding programs and the composition of environments that define the TPE of the breeding program and to study the potential effects of climate change (Figures 1C, 2–4; Ceccarelli, 1989, 1994; Cooper and Hammer, 1996; Podlich et al., 1999; Blum, 2011a; Xiong et al., 2021). The third form of the Breeder’s equation given in Figure 1C provides a partition of the heritability and predictive accuracy components of the first (Figure 1A) and second (Figure 1B) forms, to explicitly quantify trait prediction accuracy, based on the training data sets that can be created from different combinations of breeding multi-environment trials and controlled-environment facilities, and the trait performance of genotypes within the TPE (Figure 4; Cooper et al., 2021a; Diepenbrock et al., 2022; Messina et al., 2022c). In this case, the genetic correlation between the trait values observed in a multi-environment trial and the TPEs can be considered as a framework to evaluate the predictive accuracy based on the G2P model for the trait constructed in the training data sets obtained from the multi-environment trials and the true values required for trait performance in the TPEs. An example based on the schematic of the genotype reaction-norms is depicted in Figure 3A (shown as a response surface within the insert). In this example as the frequency of the water-limited environment-type E1 indicated for Environment 1 varies between a proportion of 0 and 1 within the multi-environment trial and within the TPEs, the genetic covariance component between the measurement of trait performance in the multi-environment trial and the TPE can vary between negative and positive values. This third form of the breeder’s equation can be applied as a framework to investigate the implications of breeding under current and projected future environmental conditions to evaluate the implications of breeding for drought tolerance and climate resilience under the influences of climate change. The third form of the breeder’s equation thus provides a foundation for explicit considerations of both prediction accuracy within the different possible training data sets used to construct G2P models for prediction and also the alignment of the training data sets with the TPE (Figure 1C; Cooper and DeLacy, 1994; Podlich et al., 1999; Cooper et al., 2014a, 2014b; Resende et al., 2021). As such the third form of the breeder’s equation can be used to assist breeding program design for current and future structures of the TPE to consider many influences of climate change on rates of genetic improvement for climate resilience (Figure 3; Chapman et al., 2012; Cooper et al., 2021a, 2022; Messina et al., 2022a, 2022c).

The concepts of envirotyping and environmental characterization, to provide a foundation that enables physiological interpretations of GxE interactions, have a long history in plant breeding (Knight, 1970; Byth and Mungomery, 1981; Blum, 1988, 2011a; Cooper and Hammer, 1996; Chapman et al., 2000; Chenu et al., 2011; Xu, 2016; Van Eeuwijk et al., 2016; Cooper and Messina, 2021; Costa-Neto et al., 2021; Gage et al., 2021; Li et al., 2021; Resende et al., 2021, 2022; Piepho, 2022). The details, methods, and potential applications of the characterizations have advanced with measurement technologies and the development of specialized controlled-environment and field-based research facilities (Figures 2–4; Blum, 1988, 2011a; Cooper et al., 1995, 2014a, 2014b; Rebetzke et al., 2013; Araus and Cairns, 2014; Vadez et al., 2015; Gage et al., 2021; Li et al., 2021; Smith et al., 2021b; Washburn et al., 2021; Langstroff et al., 2022). Early environmental descriptors were based on the mean grain yield of the genotypes included within experiments (Finlay and Wilkinson, 1963; Allard and Bradshaw, 1964; Eberhart and Russell, 1966; Knight, 1970). In one coarse-grained refinement, breeders distinguished between favorable environment-types and stress-impacted environment-types (Blum, 1988, 2011a; Gaffney et al., 2015; Cooper and Messina, 2021; Welcker et al., 2022). Such coarse-grained descriptors, including abiotic and biotic factors, are frequently used to distinguish among types of stress environments. However, on-farm environments tend to be mixtures of these different stress types, with the dominant stress type changing during the crop lifecycle.

**Figure 5:**
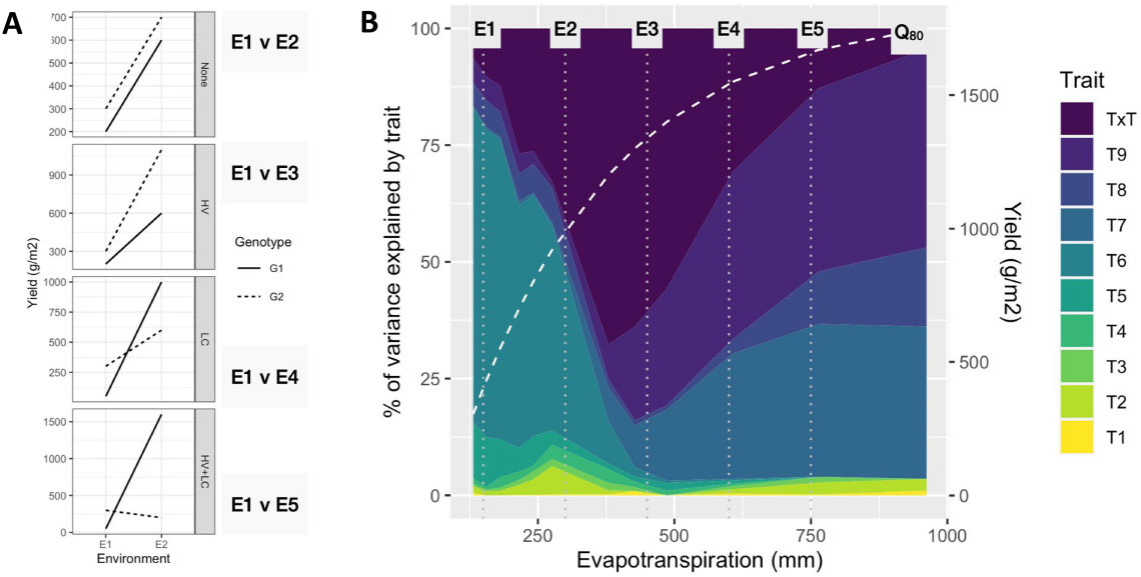
Schematic representations of GxE interactions. A, The emergence of GxE interactions for grain yield and two contrasting genotype reaction-norms (G1, G2) for grain yield along a continuum of environments (E1–E5) varying for crop water availability, as determined by crop evapotranspiration (Messina et al., 2022a, 2022b). B, The grain yield variation reaction-norms along the water availability continuum are an outcome of changes in the contributions of different physiological processes and traits (T1–T9) and trait networks (indicated by the different trait contributions and the TxT interactions) during the crop lifecycle that determine the grain yield outcomes as the environmental conditions change. Yield is modeled as a function of GxExM conditions along the water availability continuum. The continuum of water availability can be quantified by applying appropriate environmental descriptors as demonstrated in Figure 3 and described in Figure 4. The 80% quantile yield-evapotranspiration front (Q80) from Figure 3 is superimposed to indicate how different trait combinations are expected to contribute to the grain yield performance of the maize hybrids along the environmental continuum of water availability. The combination of different trait contributions and genetic variation for the traits within the reference population of genotypes under improvement by the breeding program contributes to the emergence of the genetic variation for grain yield, GxE interactions between environment types that were identified by envirotyping (indicated for comparisons between environment type E1 and environment types E2–E5 along the water availability continuum), and the contrasting grain yield reaction-norms indicated for the two hybrids. For reference, Figure 2 provides an empirical demonstration of examples of contrasting maize hybrid grain yield reaction-norms for a stratified sample of contrasting environment types.

Advances in proximal and remote sensor methodology for spatial and temporal measurements of important environmental variables have enabled refinements in the level of resolution and deconvolution of some of the combinations of abiotic and biotic environmental variables that contribute to GxE interactions throughout the crop lifecycle (Figure 4; Cooper et al., 2014a, 2014b, 2020; Costa-Neto et al., 2021; Gage et al., 2021; Li et al., 2021; Smith et al., 2021a; Washburn et al., 2021; Diepenbrock et al., 2022; Messina et al., 2022a, 2022c; Piepho, 2022). To target breeding efforts for stresses such as drought, investigations have been undertaken to quantify the occurrences of repeatable GxE interactions for yield and the contributions from traits contributing to improved yield stability across drought-affected environments (Figures 2–5; Chapman et al., 2000, 2003; Löffler et al., 2005; Chenu et al., 2011; Blum, 2011a; Kholová et al., 2013; Cooper et al., 2014a; Messina et al., 2015, 2022a, 2022c; Carcedo et al., 2022). Today the availability of many drought-specific environmental predictors has created new opportunities for their incorporation within prediction models to account for repeatable components of the total GxE interaction variance for a TPE (Boer et al., 2007; Heslot et al., 2014; Jarquín et al., 2014; Millet et al., 2019; Messina et al., 2018; Costa-Neto et al., 2021; Crossa et al., 2021; Gage et al., 2021; Li et al., 2021; Resende et al., 2021; Washburn et al., 2021; Diepenbrock et al., 2022; Piepho, 2022).

## Targeted breeding for drought resistance: genotyping and prediction-based breeding

Genotyping for breeding applications refers to the characterization of DNA sequence polymorphisms among the individuals (genotypes) created within the reference population of genotypes of the breeding program (Figure 4; Cooper et al., 2014b). Many genotyping technologies have been developed and can be applied to fingerprint the genotypes created at the different stages of breeding programs (Edwards and Batley, 2010; Yuan et al., 2017; Bayer et al., 2020; Della Coletta et al., 2021). The availability of fingerprints for genotypes at all stages of the breeding program enables the construction of genomic predictors to apply either marker-assisted selection, whole-genome prediction, or combinations of both methods (Figure 4; Lande and Thompson, 1990; Meuwissen et al., 2001; Campos et al., 2004; Barker et al., 2005; Bernardo and Yu, 2007; Heffner et al., 2009; Cooper et al., 2014a, 2014b; Voss-Fels et al., 2019a). Extensions of the breeder’s equation (Figures 1 and 4) have been developed to evaluate prediction-based breeding strategies to accelerate breeding for drought tolerance and select products with desirable yield reaction-norms for the target on-farm ***(ExM)*** environments. These are then used to model breeding strategies designed to use the genotype fingerprints of individuals and the trait variation that can be accounted for based on models of G2P relationships using the genotype fingerprints as predictors (Cooper et al., 2014a, 2020, 2021a; Jarquín et al., 2014; Gaffney et al., 2015; Technow et al., 2015; Gage et al., 2021; Li et al., 2021; Varshney et al., 2021a, 2021b, 2021c; Diepenbrock et al., 2022; Messina et al., 2022a, 2022c).

## Targeted breeding for drought resistance: phenotyping

Phenotyping for breeding applications refers to the collective of activities that are focused on measurement of the plant traits as they are expressed within an appropriate target environment context, as discussed for envirotyping, for the genotypes that are under evaluation at the different stages of breeding programs (Figure 4; Cooper and Hammer, 1996; Campos et al., 2004; Hammer et al., 2009; Blum, 2011a; Araus and Cairns, 2014; Cooper et al., 2014a, 2014b; Van Eeuwijk et al., 2019; Reynolds et al., 2020; Kholová et al., 2021). The data obtained from phenotyping studies, conducted on experiments designed to expose genetic variation for specific traits, provide estimates of the parameters that are the components of the breeder’s equation (Figure 1; Buckler et al., 2009; Cooper et al., 2014a, 2014b; Wisser et al., 2019; Diepenbrock et al., 2022; Messina et al., 2022c). To enable breeding for drought resistance, trait phenotyping involves testing genotypes from the different stages of the breeding program in the ***(ExM)*** environments that expose genotypes to the range of water-limited conditions relevant to the agricultural droughts that frequently occur within the TPE of the breeding program (Figures 2–5; Blum, 2011a; Messina et al., 2011; Cooper et al., 2014a, 2014b; Gaffney et al., 2015; Diepenbrock et al., 2022; Messina et al., 2022a, 2022c; Welcker et al., 2022). Under such water-limited testing regimes, the genetic variation for yield, and the traits contributing to yield, that is exposed for selection (Figures 2–5), can be investigated and inferred to be associated with the traits that contribute to improved yield performance and the target yield reaction-norms under relevant agricultural drought conditions within the TPE. Further, targeted phenotyping for relevant traits, that have been demonstrated to contribute to improved yield under drought in the reference population of genotypes for the breeding program, can be undertaken to enhance selection for traits and trait network strategies contributing to drought resistance and accelerate genetic gain over sequential breeding program cycles (Figure 5; Messina et al., 2011, 2015, 2021, 2022a, 2022c; Cooper et al., 2014a, 2014b; Diepenbrock et al., 2022).

Tiered phenotyping approaches have been implemented to gain insights about determinants of adaptation and drought resistance (Hammer et al., 2009; Messina et al., 2011, 2015, 2021; Sinclair, 2011; Cooper et al., 2014a, 2014b). A common strategy for maize breeding imposes punctuated water deficit at flowering and grain filling, and a terminal drought treatment (Figure 2; Campos et al., 2004; Barker et al., 2005; Cooper et al., 2014a, 2014b, 2022). The combination of these stressors with phenotyping for timing of anthesis and silking, kernel set, and yield in maize enables the breeder to identify sources of genetic variation for trait networks influencing silk response to water deficit, kernel abortion, senescence/remobilization, water capture, and conservation (Figure 5; Cooper et al., 2014a, 2014b; Messina et al., 2018, 2022a, 2022c; Diepenbrock et al., 2022). Complementary phenotyping in controlled environment facilities validated the findings for silk elongation rate response to water deficit and its relationship to the anthesis–silking interval in maize (Sadok et al., 2007; Turc et al., 2016); conductance response to vapor pressure deficit (Choudhary et al. 2014; Shekoofa et al., 2015); and rooting and water uptake (Messina et al., 2021; Reynolds et al., 2021). A similar approach conducted to screen soybean genotypes in the field under water stress, verified mechanisms of water conservation, tolerance of nitrogen fixation to water deficit and yield. This enabled the breeder to recurrently use the germplasm to create drought-resistant soybean varieties (Sinclair et al., 2010).

**Figure 6:**
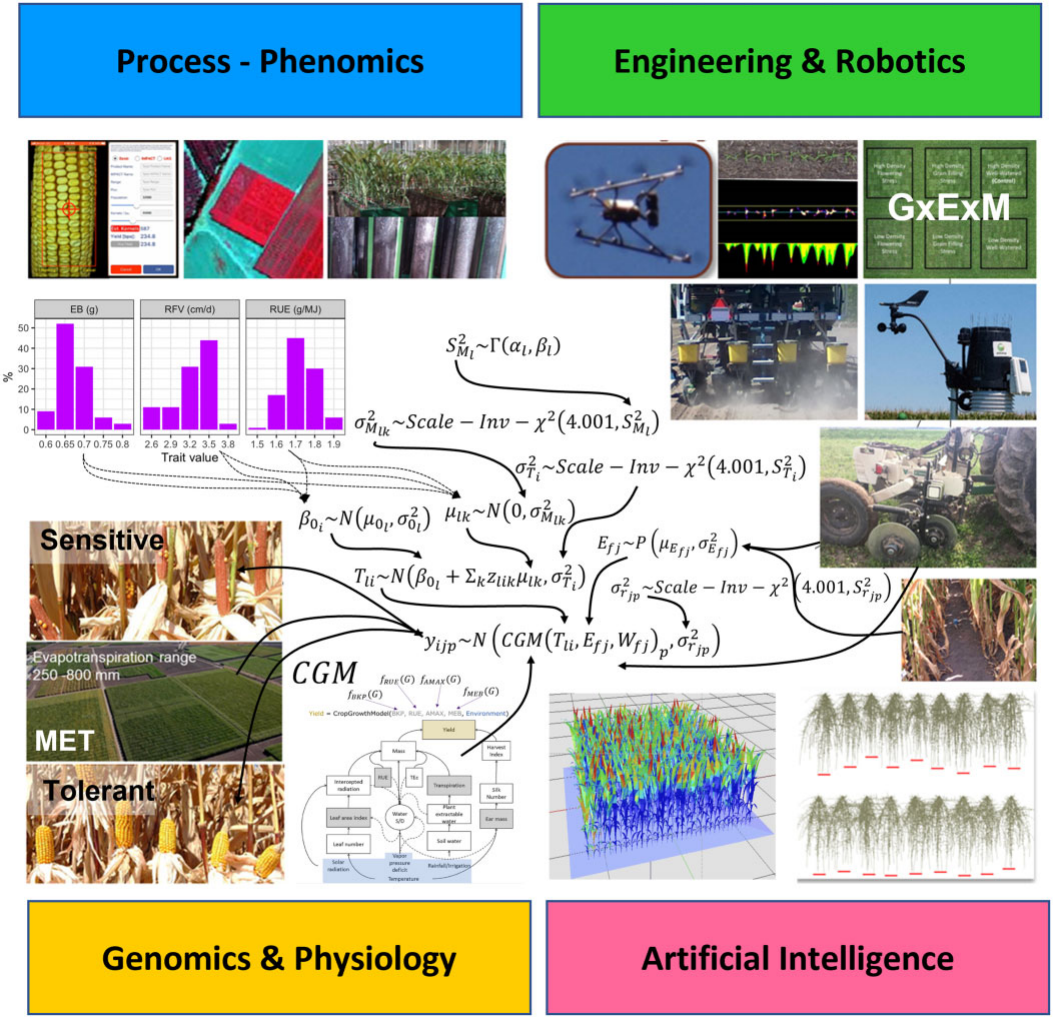
An example of the inputs from different phenotyping, envirotyping, and genotyping methods, depicted in Figure 4, used in combination with a hierarchical genotype-to-phenotype model for prediction of grain yield of maize hybrids using an appropriate crop growth model (CGM). The example is based on the application a maize CGM in combination with whole-genome prediction (WGP) for a network of traits included in the CGM (CGM-WGP) for yield prediction of maize hybrids for a range of environments that differed in water availability and is based on the studies reported by Messina et al. (2018) and Diepenbrock et al. (2022).

Phenomics is a rapidly evolving research area, enabling technology with increasing capabilities to measure the plant–crop system for multiple traits, times during the crop lifecycle, and environments (Figure 6; Araus and Cairns, 2014; Granier and Vile, 2014; Roitsch et al., 2019; Masjedi et al., 2020; Karami et al., 2021; Wang and Crawford, 2021). The coordination of envirotyping and phenotyping across stages of a breeding program thus enables evaluation of genetic variation for trait networks and ecophysiological responses to environmental variation (Figures 5 and 6; Hammer et al., 2006, 2009; Millet et al., 2019; Gleason et al., 2022; Welcker et al., 2022). This knowledge is integrated in causal G2P models for prediction to enable scaling of predictive breeding from genomes to ecosystems (Figure 6; Technow et al., 2015; Messina et al., 2018; Diepenbrock et al., 2022; Messina et al., 2022a, 2022c). Other approaches harness data and the causal connectivity of the trait networks to estimate and/or infer genetic variation for physiological traits, update the topology of the trait network based on new experimental and breeding program data, and integrate genomic and phenomic predictors simultaneously (Podlich et al., 2004; Messina et al., 2018; Van Eeuwijk et al., 2019; Diepenbrock et al., 2022; Messina et al., 2022a, 2022c). This approach led to the discovery of germplasm contributing functional genetic variation for root traits underpinning resistance to drought, residing within the reference population of genotypes of the breeding program, increased predictability of yield reaction-norms for genotypes within the TPE, and reduction of yield-gaps in water-limited environments (Diepenbrock et al., 2022; Messina et al., 2022a, 2022c).

## Gene discovery for traits: from plant cells to whole plant performance

The vision of general methods for translation of genomic information into physiological understanding and predictive methods for the expression of trait phenotypes at different scales, from cells to ecosystems, is a long-standing grand challenge for biology (Hammer et al., 2006, 2019; Band et al., 2012; Marjoram et al., 2014; Marshall-Colon et al., 2017; Ramstein et al., 2019; Peng et al., 2020; Roeder et al., 2021; Tardieu et al., 2021). While progress toward this end has been made and continues, there are still many issues to be resolved to predict the complex response surfaces of multi-trait phenomes and yield reaction-norms for the TPE of agricultural ecosystems based on the characterization of plant genomes, and to enable the ambition of prediction-based design of drought tolerant crops for current and future climates (Messina et al., 2011, 2018, 2022a; Cooper et al., 2014a, 2014b; Technow et al., 2015; Bustos-Korts et al., 2019a, 2019b, 2021; Millet et al., 2019; Ramstein et al., 2019; Voss-Fels et al., 2019a; Langridge et al., 2021; Varshney et al., 2021a, 2021b; Diepenbrock et al., 2022; Powell et al., 2022; Welcker et al., 2022; Zhao et al., 2022). Through the ongoing advances in genome sequencing capabilities to enable the investigation of trait genetic diversity within breeding populations, together with combinations of novel trait mapping studies and targeted genetic manipulation strategies, key regions of plant genomes have been identified that contain genes and natural sequence variation that underpins the expression of trait phenotypic variation at different scales (Yu and Buckler, 2006; Yu et al., 2006, 2008; Salvi et al., 2007; Buckler et al., 2009; Myles et al., 2009; Dong et al., 2012; Mace et al., 2013; Guo et al., 2014; Thoen et al., 2016; Wisser et al., 2019; Voss-Fels et al., 2019b; Bayer et al., 2020; Simmons et al., 2021; Massel et al., 2021; Tao et al., 2021; Liu and Qin, 2021; Diepenbrock et al., 2022; Tay Fernandez et al., 2022a, 2022b; Welcker et al., 2022). These genomic regions represent target entry points to further investigate and model at different scales the properties and contributions of the gene networks that are responsible for trait genetic variation and expression of phenotypic variation for trait networks in breeding populations. They also provide targets for directed manipulation to create novel genetic and phenotypic variation and potential components of the G2P models that will be required to predict the contributions of traits and trait networks to crop performance at the agricultural ecosystem level (Figure 5; Cooper et al., 2005, 2009; Hammer et al., 2006; Messina et al., 2011, 2022a, 2022c; Dong et al., 2012; Kleessen et al., 2013; Guo et al., 2014; Marjoram et al., 2014; Shi et al., 2015, 2017; Millet et al., 2019; Bustos-Korts et al., 2019a, 2019b, 2021; Ersoz et al., 2020; Massel et al., 2021; Rice and Lipka, 2021; Diepenbrock et al., 2022; Gleason et al., 2022; Powell et al., 2022; Schussler et al., 2022; Tay Fernandez et al., 2022b; Welcker et al., 2022; Zhao et al., 2022).

Significant industry-based gene discovery and expression optimization efforts have been undertaken, applying transgenic and editing-based approaches, to identify and create novel variants of genes that can positively influence crop performance for diverse water-limited agricultural environments (Castiglioni et al., 2008; Guo et al., 2014; Mall et al., 2018; Nuccio et al., 2018; Simmons et al., 2021; Linares et al., 2022a, 2022b; Schussler et al., 2022). While much has been discovered about the roles and influences of many of the genes identified and their roles in plant growth and development for a range of water-limited environmental conditions, translation of these demonstrations of biological efficacy for traits to the improvement of crop performance for the agricultural environments at the level of the TPE has been less successful, with one commercial release to date based on a drought transgene approach (Castiglioni et al., 2008; Guo et al., 2014; Shi et al., 2015, 2017; Nuccio et al., 2018; Simmons et al., 2021; Schussler et al., 2022). In their compilation of the gene targets that had been tested for yield efficacy in maize, Simmons et al. (2021) highlighted the importance of gene targets in the key hormonal pathways that are involved in regulating plant growth and development. These important hormonal pathways have many direct and indirect influences on plant growth and development, that can operate across scales from cells to whole plants, suggesting there is opportunity to use such gene targets for combined experimental and modeling research efforts to predict from the genome level to multi-trait networks that impact the yield reaction-norm phenotypes at the ecosystem level (Figure 5; Hammer et al., 2006, 2019; Powell et al., 2021, 2022; Diepenbrock et al., 2022; Gleason et al., 2022). A further promising opportunity that has been investigated is the use of these novel sources of trait genetic diversity originating from the discovery programs as components of integrated breeding strategies, where selection is targeted to co-develop complementary natural genetic diversity to exploit the positive interactions and ameliorate the potential negative consequences of the genes in different genetic backgrounds (Simmons et al., 2021; Linares et al., 2022a, 2022b).

## G2P models for traits

Large-scale, long-term research programs that have focused on breeding to improve crop drought resistance, applying combinations of forward and reverse genetics discovery methodologies that integrate linkage and association mapping approaches, together with targeted gene discovery and optimization methods, have provided deep insights into important regions of crop genomes that harbor important sources of functional, natural trait variation. These provide targets to create novel sources of G2P variation for some of the key traits and trait networks contributing to yield variation and drought resistance for agricultural environments within the reference populations of genotypes of elite breeding populations and for the genomic regions that have been under historical effects of long-term selection (Figure 5; Campos et al., 2004; Duvick et al., 2004; Guo et al., 2004, 2014; Barker et al., 2005; Feng et al., 2006; Yu and Buckler, 2006; Yu et al., 2006, 2008; Boer et al., 2007; Buckler et al., 2009; Hammer et al., 2009; Dong et al., 2012; Jordan et al., 2012; Mace et al., 2013; Morris et al., 2013; Cooper et al., 2014a, 2014b; Gaffney et al., 2015; Shi et al., 2015, 2017; Messina et al., 2015, 2018, 2022a, 2022c; Voss-Fels et al., 2019b; Wisser et al., 2019; Gage et al., 2021; Simmons et al., 2021; Varshney et al., 2021c; Diepenbrock et al., 2022; Gleason et al., 2022; Groen et al., 2022; Schussler et al., 2022; Welcker et al., 2022). Our understanding of the molecular, biochemical, and physiological processes that influence plant growth and development has expanded for model and crop plants, together with the technologies available to study the structure and function of plant genomes (Lovell et al., 2015; Thoen et al., 2016; Simmons et al., 2021). This has stimulated optimism that we can further accelerate breeding for complex challenges, such as improved crop drought tolerance, to develop more climate-resilient crops and reduce on-farm yield-gaps (Ramstein et al., 2019; Voss-Fels et al., 2019a; Peng et al., 2020; Cooper et al., 2021a; Langridge et al., 2021; Varshney et al., 2021b; Messina et al., 2022a, 2022c). Nevertheless, major gaps in our understanding of the genetic architecture and G2P relationships for traits and trait networks persist (Hammer et al., 2006; Reynolds et al., 2021; Roeder et al., 2021; Powell et al., 2021, 2022). Despite the advances, much of our prior G2P knowledge of the traits and trait networks contributing to plant growth and development has yet to be fully utilized and applied in most of the breeding programs that target improved drought resistance for agricultural environments. The challenge of targeted breeding for improved levels of drought resistance persists for most crops and regions affected by drought (Blum, 2011a; Kholová et al., 2021; Langridge et al., 2021).

## Prediction challenge to accelerate breeding for improved climate resilience: from plant cells to GxExM interactions for agricultural ecosystems

The lessons learned—successes and failures—that have contributed to the long-term progress in breeding maize hybrids with improved drought resistance for the US corn belt serve as a useful evidence-based case study against which we can judge the opportunities and challenges faced in breeding for further improvements in crop drought resistance for agricultural environments, where the frequencies of water-limited environments are expected to increase due to the effects of climate change and with any associated shifts in agronomic and agricultural system practices. First, we highlight and discuss the opportunities that have been enabled through progress toward predictive breeding methods and then consider the significant challenges that will need to be addressed to transfer the achievements for maize in the US corn belt to other crops in the same region and for maize and other crops in other global regions. Given the transdisciplinary emphasis of this review, we consider the potential for applications of mechanistic understanding of traits to target and accelerate breeding for drought resistance. An emerging opportunity we encourage is the potential to model combinations of traits as trait networks that can enable physiologists and breeders to move beyond defining static crop ideotypes to be pursued as target outcomes of breeding programs, toward a more iterative, collaborative process where the ideotypes are considered as candidate workable solutions as additional inputs to breeding program cycles. As such these inputs can augment the many other sources of inputs that are considered within breeding programs. Through this iterative approach, as the trait network targets contribute new diversity that can be exploited by the breeding programs, they then have an increased opportunity to be refined and contribute to the progression of new products developed over multiple cycles by the breeding programs (Messina et al., 2020, 2022a, 2022c).

## Breeding for drought resistance: opportunities

While many challenges persist, successful outcomes from long-term breeding programs that have targeted improved performance in water-limited (drought) environments have been demonstrated for a range of crops, including maize (Cooper et al., 2014a, 2014b; Gaffney et al., 2015; Adee et al., 2016; Cairns and Prasanna, 2018; Prasanna et al., 2021; Messina et al., 2022a; Nurmberg et al., 2022; Welcker et al., 2022), sorghum (Jordan et al., 2012; Borrell et al., 2014; Velazco et al., 2019), and wheat (Richards et al., 2014; Langridge and Reynolds, 2021; Xiong et al., 2021; Zhao et al., 2022). These and other examples of success can be used to identify common elements that have repeatedly contributed to the successful outcomes and to identify the differences for crops and regions that required crop specific research solutions to target the breeding strategy for drought resistance.

A common thread that ties successful programs delivering drought tolerant crops is selection for traits occurring at the same level of organization as the intended outcome (yield reaction-norm at the crop level; Figure 3) or at the immediate lower trait level with a clear understanding of how selection for the target traits would generate the desired outcome at the crop yield reaction-norm level (Figures 5 and 6); selection for longer coleoptiles in wheat enabled early planting and improved use of stored soil water (Xiong et al., 2021); selection for plants with small leaf profiles conserved water (Borrell et al., 2014); steep root angles enabled improved access of soil water stored in deep soil layers (Manschadi et al., 2008; Christopher et al., 2013; Borrell et al., 2014; Diepenbrock et al., 2022); limited transpiration changed crop patterns of water use through the crop lifecycle and enabled increased water use during reproductive stages of development (Cooper et al., 2014a, 2014b; Gaffney et al., 2015; Messina et al., 2015, 2022a, 2022c); and increased synchrony in pollination and reduced anthesis–silking interval increased reproductive resilience (Duvick et al., 2004; Cooper et al., 2014a, 2014b; Messina et al., 2019, 2021; Nurmberg et al., 2022). Maintaining this approach and selecting at the integrated trait and yield performance levels >150 maize hybrids with improved drought resistance and yield reaction-norms were commercialized over a decade of breeding in the USA and Brazil (Gaffney et al., 2015; Messina et al., 2022a; D. Bubeck, personal communication). Although biological efficacy was demonstrated for many genes, in particular for those implicated in the ethylene pathway, to date only one family of maize hybrids was created using a transgenic approach (Castiglioni et al., 2008; Shi et al., 2015, 2017; Nuccio et al., 2018; Simmons et al., 2021; Schussler et al., 2022).

While we can invoke the argument that our incomplete mechanistic understanding of biological determinants of drought resistance has limited our success in using transgenic approaches to create drought tolerant crops, there is merit in considering an alternative or at least complementary hypothesis, whereby the limited success is due to emergent behaviors leading to what we refer to as emergent phenotypes; these cannot be easily predicted through synthesis and modeling of mechanistic knowledge (Anderson, 1972; Peccoud et al., 2004; Powell et al., 2022). Emergent behavior is ubiquitous in non-linear dynamical systems (Feldman, 2019), including trait G2P relationships (Messina et al., 2011; Technow et al., 2021; Powell et al., 2022). Upward and downward causation determine how genes regulate the system (Roeder et al., 2021). For example, at the crop level, photoreceptors sense neighboring plants within crop canopies and transduce signals that influence dry matter allocation at the individual plant level, and consequently determine reproductive success or failure at the organ level; this is one example of downward causation (Smith, 2000). Aquaporin activities in the root system (organ level) can have a large effect on transpiration at the canopy level of organization; this is an example of upward causation. The effect of water transport through a membrane generates staygreen phenotypes and modifies the seasonal water balance and nitrogen fixation in such a way to determine yield response under water deficits; a restriction to water flow increases yield under water deficit conditions. This is an emergent phenotype that is without doubt counter intuitive (Sadok and Sinclair, 2010; Sinclair, 2011; Cooper et al., 2014b). In contrast, reducing photorespiration in C3 plants does not scale from leaf to plant to canopy as it could be expected from an analysis of the function of the parts in isolation (Hammer et al., 2019). Interactions within and between levels of organization in space and time, and the shared control of the crop system at different levels of organization lead to amplification and dampening of signals from gene to trait phenotypes underpinning drought resistance strategies and yield performance in agricultural ecosystems (Hammer et al., 2006, 2019).

## Beyond ideotypes: seeking multiple workable solutions for the effective use of water

Mechanistic dissection of the plant and crop physiology underpinning consistent improvements in yield and yield stability of crops for water-limited environments frequently reveals the importance of coordinated networks of traits contributing to drought tolerance and yield in water-limited environments (Figure 5; Messina et al., 2011, 2020, 2021, 2022a, 2022c; Borrell et al., 2014; Tardieu et al., 2021; Gleason et al., 2022; Tardieu, 2022; Welcker et al., 2022). The integrated behavior and predicted consequences of such discovered natural and novel variation for multiple traits over levels of the biological hierarchy, from cells to crop canopies, can be evaluated for their potential contributions to breeding program outcomes. We encourage consideration of trait network targets as inputs to breeding programs, to be collaboratively evaluated and refined over multiple breeding cycles, rather their more traditional use to define crop ideotypes that are to be targeted as outcomes from a breeding program. Such collaborative refinement of the trait network targets that underpin water-use patterns through the crop development cycle, reproductive resiliency, and canopy level radiation use efficiency all contributed to the improvements in drought resistance of maize hybrids for the US corn belt (Figure 6; Cooper et al., 2014a, 2014b; Messina et al., 2020, 2022a, 2022c). An important lesson from the development of the improved hybrids over multiple breeding program cycles was that the original trait network breeding targets underwent significant modification and refinement through integrated evaluation within the breeding program. The successful maize hybrids eventually released from the breeding program possessed trait combinations that were different from the original drought resistance targets (Hammer et al., 2009; Messina et al., 2015, 2021, 2022a; Reyes et al., 2015).

While retrospective analyses are insightful, the complexity of the system makes prospective analyses and prediction difficult; an increased mechanistic understanding of the system does not necessarily imply an increased capacity to predict system outcomes after recombining the parts, for which function was understood in isolation (Anderson, 1972; Peccoud et al., 2004; Messina et al., 2011, 2019; Simmons et al., 2021; Technow et al., 2021; Powell et al., 2022). The great success in understanding molecular mechanisms underpinning drought tolerance was not correlated with an improved capacity to create successful crop ideotypes capable of significantly improving drought tolerance and yield reaction-norms in different genetic and environment contexts (Donald, 1968; Martre et al., 2015; Rötter et al., 2015; Simmons et al., 2021). The study of yield–trait performance landscapes demonstrated these are often complex, rugged surfaces to be explored by the breeding program, and thus create multiple opportunities to improve crop drought resistance and yield reaction-norms over multiple breeding program cycles (Figure 5; Cooper et al., 2005; Hammer et al., 2006; Messina et al., 2011; Technow et al., 2021). These studies also demonstrate that multiple trait pathways can create temporal dynamics at a crop level, which are all conducive to drought resistance; stomatal conductance response to vapor pressure deficit (Sinclair et al., 2010; Choudhary et al., 2014), xylem conductance (Passioura, 1972; Richards and Passioura, 1989), plant size (Borrell et al., 2014), and propensity to tillering (Wang et al., 2020) can all lead to similar patterns of water use pre- and post-flowering. Agronomic management practices such as changing planting density can further affect the soil water dynamics in ways that amplify or dampen the impacts of any trait or trait combinations (e.g., Messina et al., 2011, 2015, 2021; Cooper et al., 2023). Cropping ecosystems contexts can further determine the impact and relevance of any trait network (Figure 5; Cooper et al., 2021a, 2021b; Gleason et al., 2022; Messina et al., 2022a, 2022c).

Complexity and emergent phenotypes conspire against the concept of crop ideotypes that are constructed based on partial knowledge and often ignore the temporal dynamics of the crop system at the agroecological level. Uncertainty in climate predictions, and gene-to-trait models further complicates the use of crop models for ideotype design for climate resilience (Ramirez-Villegas et al., 2015). It has been argued that breeding crops for complex environmental challenges, such as drought, often occurs with a level of complexity operating at the “edge of chaos” (Messina et al., 2011; Roeder et al., 2021; Technow et al., 2021). An example of this interpretation was inferred from the frequent emergence of barren maize plants at high plant densities within water-sufficient environments (Daynard and Muldoon, 1983; Edmeades and Daynard, 1979; Roeder et al., 2021); resource limitations, such as occur under drought, can shift this edge across the different environments within the TPE. Therefore, one can postulate the need for several alternative crop ideotypes based on different trait networks, or multiple-workable solutions, for a well-defined mixture of target environments, which could be tested within the breeding programs (Cooper et al., 2005; Messina et al., 2011; Technow et al., 2021). Figure 5 shows how the importance of plant traits, and their combinations can change along a transect of environments along a water availability gradient within a TPE (Messina et al., 2020, 2022c; Diepenbrock et al., 2022). The complexity of resolving the trait networks underpinning yield determination is highest at intermediate stress levels along the water gradient and becomes simpler toward the extreme environments of the gradient; traits underpinning growth such as radiation use efficiency, plant size, leaf nitrogen content contribute the largest to genotypic variability for yield in the favorable, water-sufficient environments, while traits such as growth maintenance and reproductive success are most important under water-limited environments where the plants experience severe stress (Messina et al., 2019, 2022b; Gleason et al., 2022). At intermediate levels of water-deficit and stress, the proportion of yield variation explained by individual traits is low. These transitions in trait network complexity underpinning yield variation and genotype yield reaction-norms across environments lead to the emergence of different patterns of GxE interactions for yield, some of which may impose limits to what can be achieved in breeding single crops for climate resilience for the TPE of agricultural systems under current conditions and expected future conditions under climate change scenarios (Figures 3–6; Messina et al., 2020, 2022c; Diepenbrock et al., 2022).

## Breeding for climate resilience: challenges

While progress has been and continues to be made for some crops and geographies, as discussed above, breeding crops for improved levels of drought resistance that translate to improved yield reaction-norms within the on-farm TPE of agricultural ecosystems is already complex. The design of breeding programs to address improved crop climate resilience is motivated by multiple influences, including improved sustainability and nutritional security ambitions in response to the combined influences of the anthropogenic drivers of climate change and consequent increases in climate variability, projections of elevated frequencies of abiotic and biotic stress events impacting crop yield variability and increasing global population, and their intersection in regions where conditions of endemic poverty, hunger, and low food security persist (Lobell et al., 2009; Chapman et al., 2012; Van Ittersum et al., 2016; Rodell et al., 2018; Ceccarelli and Grando, 2020a; Binns et al., 2021; IPCC, 2021; Kholová et al., 2021; Prasanna et al., 2021; https://sdgs.un.org/goals). It is recognized that any improvements in climate resiliency of crops must be achieved without additional harmful, and hopefully reduced, effects of agricultural systems that are currently contributing to environmental degradation and depletion of the global freshwater resources (Rosegrant et al., 2009; Brummer et al., 2011; Rodell et al., 2018; The Rockefeller Foundation, 2021; Messina et al., 2022a, 2022c). There has been an increase in attention to accelerated breeding of crops for improved abiotic and biotic stress resistance in response to the projected increases in the frequency of elevated temperatures and changes in rainfall patterns for many agricultural regions (Chapman et al., 2012; Harrison et al., 2014; Lobell et al., 2015; Challinor et al., 2016; Hammer et al., 2020; Langridge et al., 2021; Zhao et al., 2022). While these converging pressures have combined to increase the awareness and urgency of climate resilient crops for the future, the fundamental requirements, as discussed herein, for successful improvements in sustainable crop productivity have not changed.

Achieving improvements in crop adaptation for any TPE is an ongoing process to deal with shifts in production risk due to climate variability (Howden et al., 2007; Chapman et al., 2012; Rippke et al., 2016; Atlin et al., 2017; Snowdon et al., 2020). Because adaptation of agriculture to climate change calls for adoption of improved genotypes, we should consider breeding as a process resulting from a system capable of dynamically creating new genotypes adapted to the new environments as the climate changes and with it the mixture of environments of the TPE for a given geography (Howden et al., 2007; Ceccarelli et al., 2010; Chapman et al., 2012; Harrison et al., 2014; Challinor et al., 2016; Atlin et al., 2017; Ramirez-Villegas et al., 2018, 2020; Snowdon et al., 2020; Horton et al., 2021). In some circumstances, the substitution of crops and/or changes in agronomic practices may be necessary (Rippke et al., 2016; Hunt et al., 2019; Irmak et al., 2019; Griffiths et al., 2022). We have discussed biological tradeoffs (e.g. water for carbon) and the emergence of complex GxE interactions that are expected to set limits to the creation of improved products adapted to all climates (Figure 5). Lack of correlation of genotype performance across the different environment types within a TPE is frequently the type of GxE interaction that it is most pervasive in breeding crops for water-limited environments (Cooper and Hammer, 1996, Cooper et al., 2021a; Figures 3 and 5). The interpretation of this type of GxE interaction in the context of breeding for climate change is that cultivars adapted to current climates will be maladapted to future climates; but the opposite can also be true and thus creates a need to align the pace of creation of new genotypes with modified trait network combinations with the rate of change in the environment in response to climate change (Figures 3 and 5; Chapman et al., 2012; Atlin et al., 2017; Snowdon et al., 2020; Cooper et al., 2021a; Gleason et al., 2022). Adaptation to climate change thus becomes a dynamical process whereby five conditions can determine our capacity to deliver genotypes adapted to new environments: (1) initial position of a geography within the environmental gradient that is suitable for agriculture (Figure 3); (2) the trait network complexity underpinning genotypic variation for yield and yield reaction-norms and adaptive traits at that position in the environment gradient that defines the current TPE (Figure 5); (3) how trait network complexity changes within the segment defined by the current and future TPE (Figure 5; Messina et al., 2020); (4) the rate of change in the mixture of environment-types within the TPE (Figures 3 and 5; Chapman et al., 2012; Cooper et al., 2021a); and (5) the frequency and agreement between the selection environments sampled across the stages of a breeding program and the mixture of environment-types within the on-farm TPE of the agricultural ecosystems where the breeding program products will be grown (Figures 1C, 3, 4, and 5; Cooper and Hammer, 1996; Podlich et al., 1999; Diepenbrock et al., 2022). While weighted strategies for selection based on expected frequencies of environment type has been advocated for rice production in drought prone environments in Brazil (Ramirez-Villegas et al., 2018), a framework is needed to enable adaptation to climate change through accelerated product development by breeding. Such a framework should:

● Enable a transition in mindsets where breeding objectives stem from the question: how to use genetic and agronomic levers together to maximize the societal benefit of a unit of resource use, and how to minimize environmental degradation and maximize the circularity of the production system (Rodell et al., 2018; Hunt et al., 2019, 2021; Cooper et al., 2020; Messina et al., 2022a, 2022c; Zhao et al., 2022).
● Be capable of predicting emergent phenotypes and inform how to evolve germplasm to the adjacent environment space of the future TPE in a timely manner by accounting for the expected frequencies of environment types in the selection and on-farm agricultural production situations (Figure 4; Chapman et al., 2012; Snowdon et al., 2020; Roeder et al., 2021; Messina et al., 2022a, 2022c; Powell et al., 2022).
● Enable practitioners with agricultural system platforms to harness environmental and genomic predictors within a physiological framework for many different crops and agronomy practices (Figures 4 and 6; Holzworth et al., 2014; Peng et al., 2020; Diepenbrock et al., 2022).
● Include a monitoring network to track crop adaptation to current and future climates as crops experience new environments due to anthropogenic climate change (Chapman et al., 2012; Cooper et al., 2021a).

## Conclusions: lessons learned and next steps and pathways forward

The common methodologies used to breed crops for improved drought resistance have advanced along with the technologies for studying trait genetic variation, plant genomes and phenomes, and trait G2P relationships for traits and trait networks within the context of a TPE. The systematic application of breeding strategies grounded on the foundation of the breeder’s equation (Figure 1) and the clear definitions of goals for the level of organization (e.g. Crop) and TPE (e.g. dryland and limited-irrigation Western region of the US corn belt) enabled the creation and deployment of drought resistant crops, including maize (Irmak et al., 2019; McFadden et al., 2019; Messina et al., 2022a, 2022c). At the same time, the testing of physiological and genetic hypotheses underpinning variation for adaptation within breeding programs enabled an iterative cycle, whereby new scientific knowledge was integrated within prediction frameworks (Figures 4–6) that hasten genetic gain and led to new prediction frameworks (e.g. integrating genetic gain from breeding with gap analyses to improve agronomic management strategies; Figure 3) for crop improvement (Cooper et al., 2020, 2021a, 2021b, 2023; Diepenbrock et al., 2022; Messina et al., 2022a, 2022c). Strategies focused on coordinated contributions from crop genetics and agronomic management required the creation and deployment of genotype–management (G–M) technologies to deconvolute and account for shifting GxExM interactions that impact yield and yield stability due to climate change within the agricultural ecosystem.

We have argued that breeding for improved drought resistance to enable effective use of water resources has successfully contributed to the long-term increases in crop yield and yield stability for a range of crops and agricultural systems (Duvick et al., 2004; Fischer et al., 2014; Messina et al., 2022a). Paradoxically, and arguably, the misutilization of these genetic resources within current food systems has contributed to the climate change crisis and many agricultural practices that place global freshwater resources at risk (Rodell et al., 2018). One characteristic of the “super wicked” problems we face is that at times the solutions to a problem in its current state can exacerbate the problem as it unfolds in a future state (Levin et al., 2012; Ceccarelli and Grando, 2020a). It is imperative to implement G–M technologies to improve the management of and alleviate the pressures on global freshwater resources (Rodell et al., 2018). A key question today is how to harmonize future crop breeding efforts, within and across crops, for regenerative agricultural systems that can mitigate further environmental degeneration and improve societal adaptation to climate change (Brummer et al., 2011; Ceccarelli and Grando, 2020a; Griffiths et al., 2022; Messina et al., 2022a, 2022c)? Answering this question will require building upon the quantitative genetics foundation of the breeder’s equation by developing methodologies capable of predicting emergent phenotypes to fully harness scientific discoveries in plant science from cell to on-farm fields of agricultural ecosystems (Figure 6; Diepenbrock et al., 2022; Powell et al., 2022; Messina et al., 2022a, 2022c). These methods should extend performance assessments from short-term genetic gain to include long-term impacts on the environment, use of water resources, and greenhouse gas emissions. Action now to develop platforms to facilitate the integration of scientific knowledge from genes to ecosystems, as we have discussed here, and their application within breeding programs will create new opportunities to hasten the transition toward more socially and environmentally responsible crop breeding strategies that are responsive to the pressures of climate change.

## Notes

M.C. and C.D.M. jointly conceived and wrote the manuscript.

The author responsible for distribution of materials integral to the findings presented in this article in accordance with the policy described in the Instructions for Authors (https://academic.oup.com/plcell) is: Mark Cooper (mark.cooper@uq.edu.au).
